# An Overlooked Case of Abdominal Pain and Bowel Dysfunction: Chilaiditi Syndrome

**DOI:** 10.7759/cureus.81638

**Published:** 2025-04-03

**Authors:** William Adili, Corey Mumaw, Harthik Kambhampati, Carter R Olberding, Michael Herman

**Affiliations:** 1 School of Medicine, Lake Erie College of Osteopathic Medicine, Bradenton, USA; 2 Gastroenterology, Borland Groover, Jacksonville, USA

**Keywords:** chilaiditi sign, chilaiditi’s syndrome, colonic interposition, gastroenterology medicine, general radiology

## Abstract

Chilaiditi syndrome is an uncommon and often misdiagnosed condition characterized by the interposition of the colon between the liver and diaphragm, leading to symptoms of abdominal pain, bloating, and bowel dysfunction. Risk factors include chronic constipation, anatomical variations, and diaphragmatic dysfunction. This report presents the case of a 67-year-old male with right upper quadrant discomfort exacerbated by stool retention. Imaging studies ruled out acute pathology for critical conditions but demonstrated colonic interposition suggestive of Chilaiditi syndrome. Management provided consisted of conservative measures including dietary modifications and fiber supplementation, alleviating his symptoms without necessitating invasive procedures. This report emphasizes the importance of recognizing radiographic findings and risk factors of Chilaiditi syndrome in patients with nonspecific gastrointestinal complaints to avoid unnecessary medical interventions.

## Introduction

Chilaiditi sign is a rare radiologic finding of segmental interposition of the intestine between the liver and diaphragm [1]. Chilaiditi sign carries a worldwide incidence of 0.025-0.28% on plain radiographs and 1.1-2.4% on computed tomography (CT) scans [1,2]. This finding is observed significantly more in men than women, with a 4:1 male-to-female predominance [3]. When this finding is accompanied by gastrointestinal symptoms such as abdominal discomfort, bloating, nausea, or constipation, it is termed Chilaiditi syndrome [4]. While generally benign, Chilaiditi syndrome can mimic serious conditions, including pneumoperitoneum, hepatic pathology, or diaphragmatic hernia, potentially leading to unwarranted procedures [5]. Most cases of Chilaiditi syndrome can often be managed conservatively, and surgical intervention is rarely required when correctly identified. This report highlights the case of a 67-year-old male with suspected Chilaiditi syndrome, emphasizing the importance of considering this condition in the differential diagnosis of individuals with persistent upper abdominal pain.

## Case presentation

A 67-year-old male presented to the clinic complaining of right upper quadrant abdominal pain, fullness, and persistent abdominal discomfort for several weeks. He reported that his symptoms worsened postprandially, but he denied symptoms of nausea, vomiting, weight loss, or bloody stools. Additionally, he reported a bloating sensation and occasional constipation, which he attributed to his overall level of discomfort. 

Physical examination revealed mild tenderness in the right upper quadrant without guarding or rebound tenderness. Bowel sounds were normoactive, and no palpable mass was detected. Laboratory results, including liver function tests and inflammatory markers, were within normal limits. A right upper quadrant gallbladder ultrasound was performed, and no abnormalities were observed.

**Figure 1:**
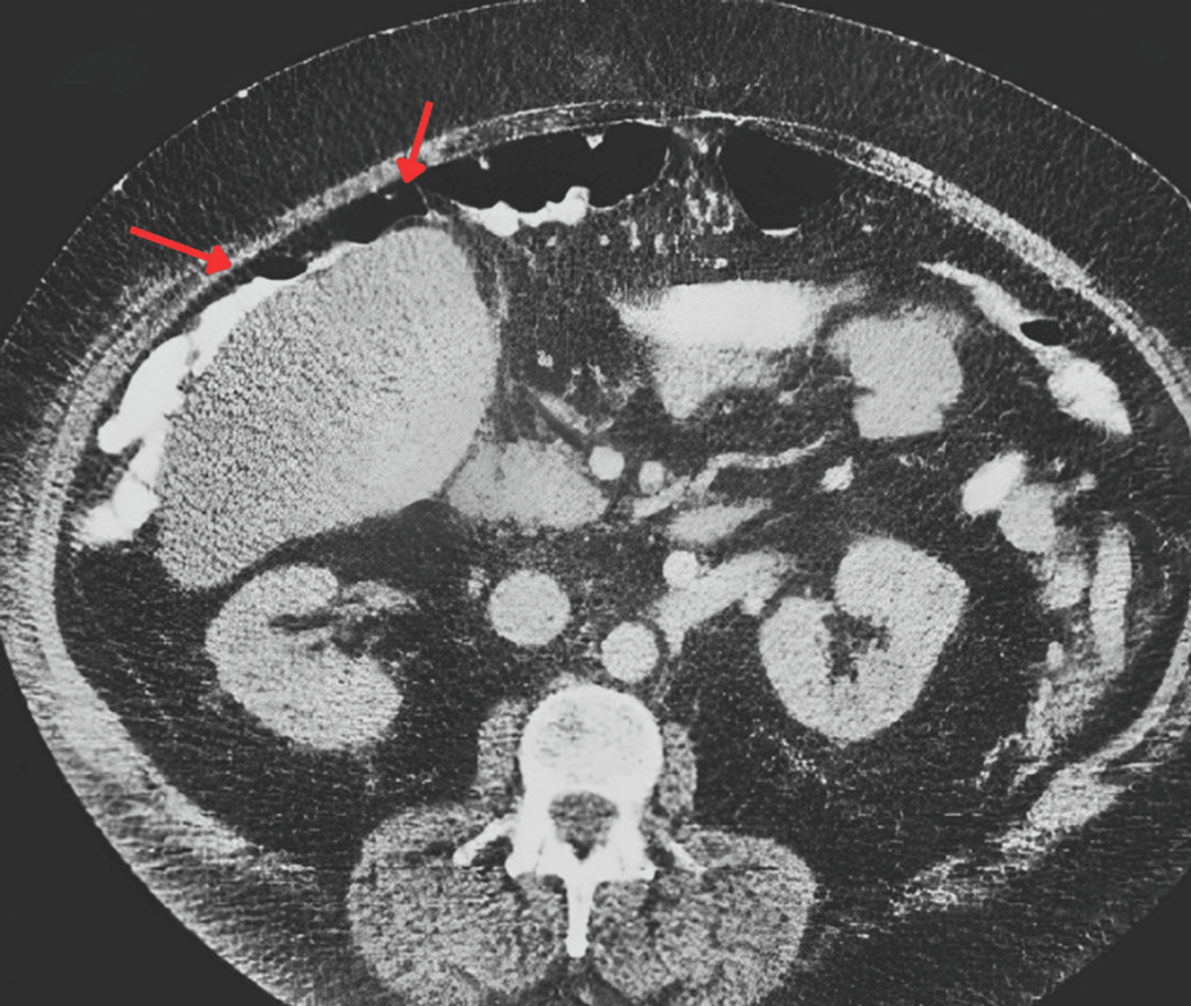
Computed tomography (CT) displaying the left colon interposition between the liver and the left diaphragm (indicated by the arrows).

CT imaging of the abdomen revealed colonic interposition between the liver and diaphragm, raising suspicion for Chilaiditi syndrome (Figure 1). Additional findings included colonic distention with stool impaction in the right colon without evidence of hepatic steatosis, free air, or significant pathology. 

## Discussion

Chilaiditi sign is often an incidental radiologic finding. However, when associated with gastrointestinal symptoms such as abdominal discomfort, bloating, and altered bowel habits, it is classified as Chilaiditi syndrome. This condition is particularly relevant in elderly patients with chronic constipation, as colonic distention and stool retention can exacerbate symptoms [6]. The reported incidence of Chilaiditi sign varies, with a greater incidence detected on CT scans (1.1-2.4%) compared to plain radiographs (0.025-0.28%), highlighting the importance of advanced imaging in its identification [2].

The pathophysiology of Chilaiditi syndrome is often multifactorial, involving anatomic variations and physiological factors. Under normal circumstances, the colon is secured by suspensory ligaments and other anatomical attachments, preventing displacement between the liver and diaphragm; however, anatomical variations, including the absence, laxity, or elongation of suspensory ligaments, can predispose to this condition [2]. Additional risk factors for Chilaiditi syndrome include chronic constipation, paralysis of the right hemidiaphragm, obesity, ascites, and cirrhosis [7]. Notably, the incidence of Chilaiditi syndrome is also higher among psychiatric inpatients, particularly those with schizophrenia, suggesting a wide range of risk factors that are not yet fully understood [8]. In this patient, chronic constipation, stool retention in the right colon, and advanced age were likely key contributors to the development and exacerbation of symptoms.

Management of Chilaiditi syndrome is generally conservative, focusing on symptom relief. This includes dietary modifications, stool softeners, and increased fiber intake to reduce colonic distention and improve bowel motility in those with constipation. Dietary recommendations include eating smaller, more frequent meals to prevent gastrointestinal overdistension, drinking at least eight glasses of water per day to promote bowel hydration and bowel motility, and limiting gas-producing foods such as legumes, carbonated beverages, and cruciferous vegetables to reduce bloating and discomfort. Surgical intervention is rarely necessary and is typically reserved for cases complicated by volvulus, ischemia, or bowel obstruction.

Recognizing Chilaiditi syndrome in patients with nonspecific gastrointestinal complaints is critical for preventing unnecessary interventions. Although a benign radiographic finding, misinterpretation of imaging findings for suspected free air under the diaphragm increases the likelihood of unwarranted exploratory surgeries, exposing patients to potential surgical complications and prolonged hospital stays. Furthermore, distinguishing Chilaiditi syndrome from other pathological conditions such as pneumoperitoneum, volvulus, or diaphragmatic hernia creates a more accurate and efficient diagnostic approach for clinicians. This increased recognition can lead to timely and appropriate management, reducing the likelihood of invasive procedures while ensuring appropriate patient care. 

## Conclusions

Chilaiditi syndrome remains an underrecognized yet important differential diagnosis for persistent abdominal pain and bowel dysfunction, particularly in elderly patients. As demonstrated in this case, chronic constipation and stool impaction can contribute to symptomatic exacerbation, best managed through dietary modifications, fiber supplementation, and bowel regulation.

Clinicians should maintain a high index of suspicion when evaluating patients with unexplained upper abdominal pain, especially if imaging reveals colonic interposition between the liver and diaphragm. Recognizing the benign nature of this condition is crucial to avoiding unnecessary interventions and ensuring effective, noninvasive management strategies to improve patient outcomes. 
